# Development and evaluation of Intensive Case Management Screening Sheet in the Japanese population

**DOI:** 10.1186/s13033-019-0278-7

**Published:** 2019-04-05

**Authors:** Kota Suzuki, Sosei Yamaguchi, Yasunari Kawasoe, Kazumi Nayuki, Tsutomu Aoki, Naomi Hasegawa, Chiyo Fujii

**Affiliations:** 1 Department of Community Mental Health and Law, National Institute of Mental Health, National Center of Neurology and Psychiatry (NCNP), 4-1-1, Ogawahigashi, Kodaira, Tokyo Japan; 2 Asahi General Hospital, I 1326, Asahi, Chiba Japan; 3 Psychiatric Day Care & Clinic Hotto Station, Odori 5, Sapporo, Hokkaido Japan

**Keywords:** Case (care) management, Community mental health, Factor analysis, Receiver operating characteristic analysis, Schizophrenia, Mood disorder

## Abstract

**Background:**

In Japan, the mental health system has been shifting from hospitalization-based to community-based care; some organizations have gradually begun providing intensive case management (ICM) services. We developed an Intensive Case Management Screening Sheet (ICMSS) to screen for the need for ICM in people with mental illness.

**Methods:**

The aim of this study was to examine the psychometric properties and discriminative ability of ICMSS. Subjects consisted of 911 people with mental illness. The ICMSS score was rated by a professional such as a nurse, social worker, or occupational therapist.

**Results:**

Exploratory factor analysis showed a one-factor structure with 14 items. The factor structure was supported by confirmatory factor analysis (comparative fit index, 0.98; Tucker–Lewis index, 0.97; root mean square error test of close fit, 0.05). In the receiver operating characteristic analysis for discriminating between users and non-users of ICM services, the area under the curve (AUC) for ICMSS was significantly larger than for Global Assessment of Functioning and Personal and Social Performance Scale, indicating better discriminative ability. However, the AUC for ICMSS was moderate. Thus, we suggest that the need for ICM services is determined by quantitative assessment (i.e., ICMSS) and clinical judgment.

**Conclusion:**

ICMSS is a brief tool for mental health professionals that will be useful in routine clinical practice. We expect that ICMSS will be used as a measure that reflects the views of professionals from various disciplines in Japanese institutions.

## Introduction

In the context of deinstitutionalization, it is widely accepted that most people with severe mental illness do not need to reside in the hospital if they receive support from community mental health services [1, 2]. Case (care) management, a method to coordinate services for meeting their needs [3], is a key component of assistance for people with mental illness to live in the community. There are several models of case management with varying levels of intensity. In broker case management, the case manager’s role is to connect people with mental illness to necessary services; but with a staff-to-client ratio of approximately 50, interventions by the case manager and outreach services are poor. Broker case management was shown to be ineffective, particularly for people with severe mental illness [4]. A case management model, which includes a staff-to-client ratio of less than 20, services directly provided by the case manager, and outreach services is generally considered intensive case management (ICM). For instance, assertive community treatment is a well-known model of ICM [5]. A Cochrane review showed that ICM services reduce hospitalization rates, increase retention in care, and improve social functioning compared to standard care [6]. However, ICM services appear to be considerably effortful and costly to provide for all patients with mental disorders; mental health resources in the real world are clearly limited. Considering the efficiency of community mental health services [7], it is imperative to assess the proper allocation of ICM services to people with severe mental illness who need them.

In Japan, the mental health system has been shifting from hospitalization-based to community-based care [8]. In addition, some organizations have gradually begun to provide ICM services. However, psychiatric hospitals and mental health clinics in Japan accept all people who potentially have mental health problems regardless of the care level needed [9]. Thus, it is important that psychiatrists and other professionals assess the need for ICM services to efficiently provide services.

People who need ICM services may be defined as those who have problems living in the community without ICM services. The current level of functioning is associated with the level of care [10]; thus, it seems to be useful as a screen for ICM need. Moreover, since ICM is effective for reducing hospitalization rates and improving social functioning [6], ICM is considered necessary for patients at risk of frequent readmissions and challenges in adapting to living in the community. Systematic reviews have pointed out that medication adherence and family involvement are factors associated with readmission [11, 12]. Prisoners, inmates, and probationers frequently have mental illness [13], and recidivism is high in people with and without mental illness [14]. Previous reviews have pointed out that poverty might cause mental illness, and mental illness worsens socioeconomics status [15, 16]. Therefore, we hypothesized that the need for ICM services is based not only on current functioning, but also on these predictors of readmission and future challenges, i.e., medication adherence, family involvement, risk of criminal activity, and financial problems.

Assessment of functioning and prediction of readmission and future challenges in people with mental illness rely on multidisciplinary assessment by psychiatrists, mental health nurses, social workers, and occupational therapists. Such assessments are made in different contexts. Multidisciplinary professionals such as nurses, social workers, and occupational therapists are involved in the care of people with mental illness under different conditions [17]. For example, nurses in general have many opportunities to interact with and observe people in medical settings [18]. Social workers help their patients receive social and community services [19]; thus, they usually have information about family, financial, and social circumstances. Occupational therapists may support their patients in acquiring occupational and daily skills [20]; they usually assess functioning and characteristics related to occupational experiences and daily activities through their clinical practice. In summary, the views of multidisciplinary professionals can shed light on multiple aspects of people with mental illness that might be helpful for identifying the need for ICM services.

Over the past three decades, several scales have been developed to assess levels of care and functioning [21–25]. The Global Assessment of Functioning (GAF) [21] and Personal and Social Performance Scale (PSP) [22] are common tools measuring functioning in people with mental illness. Lyons et al. [23] developed the Severity of Psychiatric Illness Scale to assess the need for psychiatric admission. These measures do not include all of the factors associated with the need for ICM services, since they were not specifically created for assessing this need. Furthermore, algorithms for determining levels of care were proposed in previous studies [26, 27]. The Level of Care Utilization System for Psychiatric and Addiction Services (LOCUS) was developed by the American Association of Community Psychiatrists [26] and used by some Japanese institutions [28]. LOCUS consists of six dimensions of five detailed grading systems. It classifies people with mental illness into six levels, from outpatients via ICM to inpatients [26]. Raters need training to precisely evaluate people with mental illness according to the five detailed grading systems. In addition, if clinical staff members use LOCUS, they may need much effort to gather the needed information. In other words, it may be hard for professionals in various disciplines to acquire the relevant information in routine clinical practice to use LOCUS. Taken together, for the Japanese mental health system, we considered it important to develop a scale that comprehensively includes factors associated with the need for ICM and is easy for professionals from various disciplines to rate.

In this context, we developed the Intensive Case Management Screening Sheet (ICMSS) to briefly assess the need for ICM services in people with mental illness. ICMSS consists of items relevant to current functioning and predictors for readmission and future challenges in living independently. In addition, we developed ICMSS for professionals from various disciplines to use easily in clinical practice. Using data from two institutions that voluntarily provided ICM services to people with mental illness [29, 30], we aimed to examine the psychometric properties of ICMSS and its discriminative ability for users and non-users of ICM services.

## Methods

### Participants

**Fig. 1:**
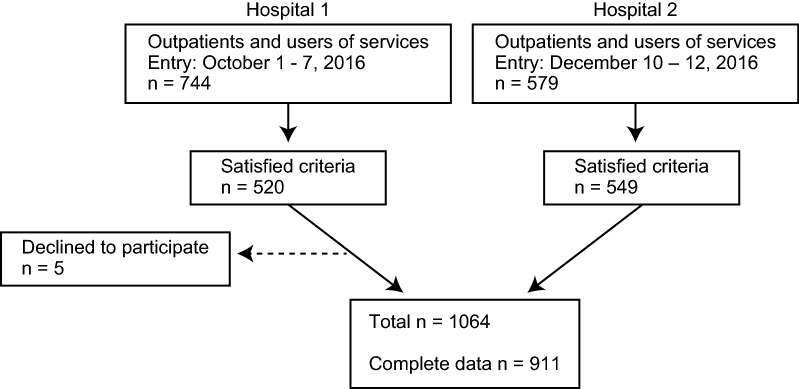
Study flowchart

Participants were recruited from a population of patients who received psychiatric outpatient services, psychiatric day care, or outreach services at two medical institutions during the study entry period (Fig. 1). We set the following inclusion criteria for this study: age over 20 years; continuous use of services for at least 6 months at one of the participating medical institutions or a total duration of mental health services use of at least 12 months; and diagnosis codes from F10 to F99 in the International Statistical Classification of Diseases and Related Health Problems, 10th revision. We excluded patients who utilized residential care facilities for the aged and disabled, except for group home care. In terms of informed consent, we employed an opt-out approach in this study. Participants were provided information about this study via posters about the study in waiting rooms, which guaranteed opportunities to decline participation in the study and the use of their data.

A total of 1064 people participated in this study (Fig. 1). We used data from 911 people in the analysis after exclusion of missing values. ICM services were provided to 309 of 911 people at two institutions. The characteristics of each group are shown in Table 1. The study protocol was approved by the ethics committee of Asahi General Hospital (No. 2016092001).

**Table 1:** Characteristics of participants (*n* = 911)

	ICM	Non-ICM	*P* value
N	309	602	
*Age*			
Mean	45.09	48.93	< 0.001
SD	12.48	16.11	
*Sex*			
Male (%)	59.87	46.51	< 0.001
*Diagnosis*			
Schizophrenia spectrum disorder (%)	46.60	26.74	< 0.001^a^
Mood disorder (%)	20.71	40.03	< 0.001^a^
Other (%)	32.69	33.22	NS^a^
*Physical problem*			
%	17.15	15.95	NS
*Previous hospitalization*			
%	20.39	11.46	< 0.001
*GAF*			
Mean	54.23	59.08	< 0.001
SD	14.70	14.35	
*PSP*			
Mean	57.08	63.21	< 0.001
SD	14.49	15.52	

*GAF* Global Assessment of Functioning, *PSP* Personal and Social Performance Scale, *NS* nonsignificant

^a^Bonferroni correction

### Intensive Case Management Screening Sheet (ICMSS)

ICMSS was preliminarily developed based on similar research projects regarding case management. First, 11 items were created to assess the need for ICM services in acute psychiatric units [31]. Next, 19 items were created by modifying the original 11 items with reference to the Discharge Difficulty Scale [32] for a study involving inpatients [33]. These items were used for other Japanese studies [34]. Four of 19 items were associated with issues related to hospital admission; these items were excluded for this study. Consequently, ICMSS consisted of 15 items related to current functioning and predictors for readmission and future challenges in living independently.

Mental health professionals (e.g., social workers, nurses, and occupational therapists) evaluated participants using ICMSS. They rated each item as yes, no, or unknown. There were unknown responses for 196 of 911 participants. For factor analysis, 196 participants were excluded from the dataset (i.e., *n* = 715). On the other hand, we considered it useful to group unknown and missing responses together for ICMSS scoring; such scoring procedures have been used in other clinical assessment tools, e.g., Camberwell Assessment of Need [35]. Thus, we performed ROC analysis using both datasets with 715 and 911 participants, respectively.

### Variables

Attending doctors rated the participants’ functioning using Japanese versions of GAF and PSP. All doctors had received training on the rating methods for these scales. GAF is a measure of social, occupational, and psychological functioning [36]. PSP consists of four domains of personal and social functioning, including socially useful activities, personal and social relationships, self-care, and disturbing and aggressive behavior [22]. Scores for each measure range from 1 to 100, with higher scores representing better functioning.

### Analysis

Statistical analysis was performed with R 3.3.1 [37] and the “psych,” “polycor,” “MASS,” “pROC,” “epiDisplay,” and “lavaan” packages. The dataset (*n* = 715) was divided into two subsets for exploratory and confirmatory factor analyses using the random function. The minimum average partial correlation (MAP) test was performed using a polychoric correlation matrix to determine the factor structure. We performed exploratory factor analysis with polychoric correlation analysis and maximum likelihood estimation, because values for each ICMSS item were either zero or one. For confirmatory factor analysis, we used a diagonally weighted least squares method. All of the data (*n* = 715) were used for correlational analyses. Pearson’s correlation coefficients between measures were calculated. Using both datasets (*n* = 715 and *n* = 911), we performed logistic regression on discriminating between users and non-users of ICM services and ICMSS, PSP, age, sex, previous hospitalization, diagnosis of schizophrenia spectrum disorder (F20–29), and diagnosis of mood disorder (F30–39). Stepwise selection was performed based on the Akaike information criterion (AIC).

## Results

### Factor analysis

MAP was smallest for a one-factor model (one factor, 0.016; two factor, 0.021). Thus, we considered the one-factor model appropriate for ICMSS. Table 2 shows the results of the exploratory factor analysis. We excluded one item with low factor loadings (0.22): “The latest hospitalization was involuntary.” Factor loadings for other items were greater than 0.35. Confirmatory factor analysis (Table 2) showed excellent fit (comparative fit index, 0.98; Tucker–Lewis index, 0.97; root mean square error test of close fit, 0.05), while the *χ*^2^ test was significant (*χ*^2^(77) = 148.61, *P* < 0.01). In addition, Cronbach’s α showed acceptable internal consistency (*α* = 0.77).

**Table 2:** Factor loadings in exploratory factor analysis (EFA) and confirmatory factor analysis (CFA) for the Intensive Case Management Screening Sheet (ICMSS) (*n* = 715)

	EFA	CFA
2. He/she has had serious problems with performing tasks necessary for community life independently, such as management of nutrition, sanitation, money, safety, human relations or document, and movement (including cases with overburdened families)	0.87	0.87
6. He/she has committed violence against, insulted, or refused a relationship with his/her family members	0.80	0.67
10. His/her knowledge or understanding about his/her illness is insufficient, or he/she has poor understanding of his/her treatment	0.80	0.80
7. He/she has experienced an intervention by police or public health officials	0.78	0.74
3. He/she has committed violence against non-family members, property damage, and behaved in an annoying manner or had trouble with neighbors	0.77	0.79
12. He/she has had financial problems such as with purchasing of daily necessities and payment for utilities or medical expenses	0.76	0.88
4. He/she has the experience of being missing, losing a residence, being threatened with eviction, or becoming homeless	0.72	0.90
8. He/she could not take medicine regularly for more than 2 months. (Choose “No” if it is the first time that he/she has used psychiatric services)	0.72	0.79
1. He/she has had a serious problem in performing social roles (employment, school attendance, domestic labor) for 6 months continuously	0.71	0.71
13. He/she has had financial problems with paying rent	0.67	0.97
5. He/she has attempted self-harm or suicide	0.5	0.51
9. He/she has not visited the outpatient clinic for more than 2 months. (Choose “No” if it is the first time that he/she has used psychiatric services)	0.49	0.59
14. He/she has no family members to support him/her (for example, refusal or non-cooperation from family or no relatives and solitude)	0.47	0.48
15. Family members living together have difficult problems (such as nursing care, poverty, education, disability) that require assistance	0.36	0.16
11. The latest hospitalization was an involuntary admission	0.22	NA

*NA* not available

### Correlational analyses

ICMSS was significantly correlated with GAF (*r* = − 0.48 < 0.001). We also found the significant correlation between ICMSS and PSP (*r* = − 0.53 < 0.001).

### ROC analyses

**Fig. 2:**
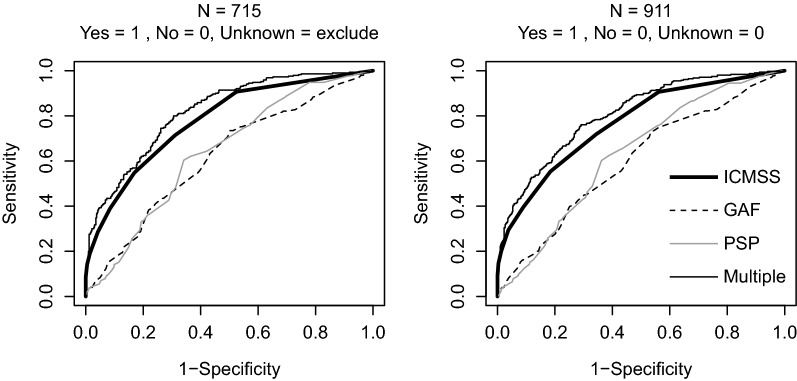
Receiver operating characteristic (ROC) curves for Intensive Case Management Screening Sheet, Global Assessment of Functioning (GAF; reversed) and Personal and Social Performance Scale (PSP; reversed) using two datasets with 715 and 911 participants. Multiple represents the result of the logistic regression analysis shown in Table 4

Figure 2 shows the ROC curves for ICMSS, GAF, and PSP for both datasets. The area under the curve (AUC) was significantly larger for ICMSS (*n* = 715: AUC, 0.78; 95% CI 0.75–0.81; *n* = 911: AUC, 0.77; 95% CI 0.74–0.80) than GAF (*n* = 715: AUC, 0.62; 95% CI 0.58–0.66; *n* = 911: AUC, 0.60; 95% CI 0.56–0.64) and PSP (*n* = 715: AUC, 0.64; 95% CI 0.60–0.68; *n* = 911: AUC, 0.63; 95% CI 0.60–0.67) (all ps < 0.001). For ICMSS (Table 3), the cutoff of ≥ 1 resulted in high sensitivity (*n* = 715: 0.91; *n* = 911: 0.91) and low specificity (*n* = 715: 0.48; *n* = 911: 0.44), whereas the cutoff of ≥ 2 had moderate sensitivity (*n* = 715: 0.71; *n* = 911: 0.72) and moderate specificity (*n* = 715: 0.69; *n* = 911: 0.66). We found no remarkable difference in results between the two datasets.

**Table 3:** Sensitivity and specificity for the Intensive Case Management Screening Sheet (ICMSS) by score

Scores	*n* = 715		*n* = 911	
	Sensitivities	Specificities	Sensitivities	Specificities
1	0.91	0.48	0.91	0.44
2	0.71	0.69	0.72	0.66
3	0.55	0.83	0.55	0.82
4	0.39	0.92	0.39	0.91
5	0.29	0.96	0.29	0.96
6	0.19	0.99	0.20	0.99

Table 4 shows the stepwise logistic regression analysis results. Use of ICM services was significantly associated with ICMSS, PSP, age, sex, previous hospitalization, and diagnosis of schizophrenia spectrum disorder. AUC improved in the model (*n* = 715: AUC, 0.82, 95% CI 0.79–0.85; *n* = 911: AUC, 0.80, 95% CI 0.77–0.83) (Fig. 2) and ICMSS remained significant in the model (*P* < 0.01).

**Table 4:** Results of stepwise logistic regression analysis for discriminating between users and non-users of intensive case management

	*n* = 715		*n* = 911	
	Odds ratio (95% CI)	*P* value	Odds ratio (95% CI)	*P* value
ICMSS	1.69 (1.51–1.90)	< 0.001	1.72 (1.56–1.91)	< 0.001
PSP	1.02 (1.00–1.03)	0.001	1.02 (1.01–1.04)	0.001
Age	0.99 (0.98–1.00)	0.060	0.98 (0.97–0.99)	0.004
Sex (male/female)	1.62 (1.13–2.32)	0.009	1.73 (1.25–2.40)	0.001
Schizophrenia spectrum disorder	2.06 (1.37–3.09)	< 0.001	2.13 (1.49–3.06)	< 0.001
Previous hospitalization	1.20 (1.10–1.30)	< 0.001	1.11 (1.04–1.18)	0.001

For 715 and 911 data, same variables were retained by the stepwise selection

*ICMSS* Intensive Case Management Screening Sheet, *PSP* Personal and Social Performance Scale

## Discussion

In this study, we developed the ICMSS to assess the need for ICM services among people with mental illness and examined its psychometric properties and discriminative ability. Exploratory factor analysis showed a one-factor structure and 14 items were selected by the factor loadings. Confirmatory factor analysis also supported the factor structure. ICMSS had a significantly larger AUC than GAF and PSP, indicating that it had better discriminative ability. Previous studies have identified some predictors such as medication adherence and family involvement [11, 12]. GAF and PSP are associated with current functioning, whereas ICMSS was developed to assess both current functioning and predictors for readmission and future challenges in living independently. ICM services are necessary not only to support people with current problems, but also to prevent readmission and future challenges in living independently. Thus, we suggest that ICMSS is useful for assessing the need for ICM services in people with mental illness.

For ICMSS, sensitivity was high for both datasets when the cutoff was ≥ 1, but drastically lower with a cutoff of ≥ 2. Hence, we suggest that a cutoff of ≥ 1 is appropriate for screening whether ICM services are needed. However, the AUC was not very large, even in the stepwise logistic regression analysis. In this study, ICMSS was rated by a single mental health professional on a multidisciplinary treatment team; thus, the ICMSS score for each participant does not reflect the views of professionals in other disciplines. Flexible clinical judgment seems important for decision-making about whether to provide ICM services. Therefore, we thought that the need for ICM services was being determined by quantitative assessments (i.e., ICMSS) and clinical judgment.

We performed ROC analysis to evaluate the discriminative ability of ICMSS. For one dataset (*n* = 715), we excluded unknown ICMSS responses. Unknown and missing responses were grouped together for ICMSS scoring for the other dataset (*n* = 911). Regardless of the difference in scoring, the results from the two datasets showed similar discriminative ability. Grouping unknown and missing responses appeared to be useful for clinical ICMSS scoring. Moreover, in the Camberwell Assessment of Need, which is used broadly in clinical settings, a similar scoring procedure is applied for unknown responses [35]. Although it is necessary to gather as much information about users as possible for ICMSS scoring, we may recommend grouping together unknown and missing responses for scoring in a clinical setting.

Algorithms for determining the level of care were proposed in previous studies [26, 27]. Ideally, these algorithms can be used in the Japanese system. It is expected that community mental health services, including ICM services, are provided according to actual resource and community characteristics [7]. In Japan, the mental health system is shifting from hospitalization-based to community-based care [8]. However, ICM services are not fully supported by the Japanese system; the costs of ICM services are borne by the medical institution. Thus, the criteria for providing ICM services changed flexibly according to resource and staff assignments at each institution in Japan. Algorithms provide a precise classification of the level of care, but they are inflexible. ICMSS helps in the acquisition of information by professionals of various disciplines that we consider useful for flexible judgment in Japanese institutions.

In confirmatory factor analysis, the *χ*^2^ test was significant, suggesting poor model fit. Generally, the *χ*^2^ test is sensitive to sample size and tends to reject models with a large sample. Our sample was relatively large. However, other indices showed excellent fit. Thus, we believe that a one-factor model is appropriate for ICMSS. The age range of our participants was broad (20–92 years). In the stepwise logistic regression analysis, ICMSS remained significant in the model including age. However, in Japan, persons aged > 65 years can choose to use welfare system services for elderly people, which might have affected the present results. This study included patients from two psychiatric institutions. The generalizability of findings may be limited. In addition, participants were recruited from patients who received psychiatric outpatient services, psychiatric day care, or outreach services. We did not obtain data from current and recently discharged inpatients of psychiatric hospitals. The results might vary when focusing on inpatients.

In summary, we developed ICMSS for nurses, social workers, and occupational therapists to use easily in clinical practice. ROC analysis showed that ICMSS has better discriminative ability between users and non-users of ICM services than GAF and PSP. Thus, we suggest that ICMSS is a useful screening tool that reflects the views of professionals of various disciplines. However, the AUC of ICMSS was not large. The need for ICM services might be associated with quantitative assessment (i.e., ICMSS) and clinical judgment. In Japan, ICM services are provided only by a limited number of advanced organizations. We expect that ICMSS will be used as a measure that reflects the views of professionals from various disciplines in several Japanese institutions, which may contribute to the further spread of ICM services in Japan.

## Abbreviations

ICM: intensive case management
ICMSS: Intensive Case Management Screening Sheet
AUC: area under the curve
GAF: Global Assessment of Functioning
PSP: Personal and Social Performance Scale
LOCUS: Level of Care Utilization System for Psychiatric and Addiction Services
ICD-10: International Statistical Classification of Diseases and Related Health Problems, 10th revision
